# Recombinant Bile Salt Hydrolase Enhances the Inhibition Efficiency of Taurodeoxycholic Acid against *Clostridium perfringens* Virulence

**DOI:** 10.3390/pathogens13060464

**Published:** 2024-05-31

**Authors:** Tahrir Alenezi, Bilal Alrubaye, Ying Fu, Janashrit Shrestha, Samar Algehani, Hong Wang, Rohana Liyanage, Xiaolun Sun

**Affiliations:** 1 Center of Excellence for Poultry Science, University of Arkansas, Fayetteville, AR 72701, USA; alrubaye@uark.edu (B.A.); js206@uark.edu (J.S.); algehani@uark.edu (S.A.); hxw01@uark.edu (H.W.); 2 Cell and Molecular Biology Program, University of Arkansas, Fayetteville, AR 72701, USA; 3 College of Medical Applied Sciences, The Northern Border University, Arar 91431, Saudi Arabia; 4 Department of Chemistry, University of Arkansas, Fayetteville, AR 72701, USA; rliyana@uark.edu

**Keywords:** virulence, bile salt hydrolase, cloning, gene and protein expression, secretory protein

## Abstract

*Clostridium perfringens* is the main pathogen of chicken necrotic enteritis (NE) causing huge economic losses in the poultry industry. Although dietary secondary bile acid deoxycholic acid (DCA) reduced chicken NE, the accumulation of conjugated tauro-DCA (TDCA) raised concerns regarding DCA efficacy. In this study, we aimed to deconjugate TDCA by bile salt hydrolase (BSH) to increase DCA efficacy against the NE pathogen *C. perfringens*. Assays were conducted to evaluate the inhibition of *C. perfringens* growth, hydrogen sulfide (H_2_S) production, and virulence gene expression by TDCA and DCA. BSH activity and sequence alignment were conducted to select the *bsh* gene for cloning. The *bsh* gene from *Bifidobacterium longum* was PCR-amplified and cloned into plasmids pET-28a (pET-BSH) and pDR111 (pDR-BSH) for expressing the BSH protein in *E. coli* BL21 and *Bacillus subtilis* 168 (*B-sub-*BSH), respectively. His-tag-purified BSH from BL21 cells was evaluated by SDS-PAGE, Coomassie blue staining, and a Western blot (WB) assays. Secretory BSH from *B. subtilis* was analyzed by a Dot-Blot. *B-sub*-BSH was evaluated for the inhibition of *C. perfringens* growth. *C. perfringens* growth reached 7.8 log10 CFU/mL after 24 h culture. *C. perfringens* growth was at 8 vs. 7.4, 7.8 vs. 2.6 and 6 vs. 0 log10 CFU/mL in 0.2, 0.5, and 1 mM TDCA vs. DCA, respectively. Compared to TDCA, DCA reduced *C. perfringens* H_2_S production and the virulence gene expression of *asrA1*, *netB*, *colA*, and *virT*. BSH activity was observed in *Lactobacillus johnsonii* and *B. longum* under anaerobe but not *L. johnsonii* under 10% CO_2_ air. After the sequence alignment of *bsh* from ten bacteria, *bsh* from *B. longum* was selected, cloned into pET-BSH, and sequenced at 951 bp. After pET-BSH was transformed in BL21, BSH expression was assessed around 35 kDa using Coomassie staining and verified for His-tag using WB. After the subcloned *bsh* and amylase signal peptide sequence was inserted into pDR-BSH, *B. subtilis* was transformed and named *B-sub*-BSH. The transformation was evaluated using PCR with *B. subtilis* around 3 kb and *B-sub-*BSH around 5 kb. Secretory BSH expressed from *B-sub*-BSH was determined for His-tag using Dot-Blot. Importantly, *C. perfringens* growth was reduced greater than 59% log10 CFU/mL in the *B-sub*-BSH media precultured with 1 vs. 0 mM TDCA. In conclusion, TDCA was less potent than DCA against *C. perfringens* virulence, and recombinant secretory BSH from *B-sub*-BSH reduced *C. perfringens* growth, suggesting a new potential intervention against the pathogen-induced chicken NE.

## 1. Introduction

Necrotic enteritis (NE) has recently reemerged as one of the prevalent poultry diseases. NE is estimated to cause around a USD 6 billion loss in the poultry industry yearly around the world [1]. *Clostridium perfringens*, a spore-forming and anaerobic Gram-positive bacterium, is the main pathogen of NE [2,3,4]. Coccidiosis caused by *Eimeria maxima* and *Eimeria acervulina* is one of the most important predisposing factors [5]. The increased NE incidence is the result of a restriction of prophylactic antimicrobials in poultry production [6]. Broiler chickens with either clinical or subclinical NE have inferior growth performance in terms of body weight gain and feed efficiency [7]. The absence of effective antimicrobial alternatives is becoming more and more challenging for poultry and allied industries. Several food and restaurant companies have recently delayed or backtracked from their pledge of “no-antibiotics-in-chicken”. Hence, it is urgent to develop antimicrobial-free alternatives to reduce chicken NE.

One of the emerging alternatives is bile acid intervention. Bile acids have long been mainly studied for facilitating lipid digestion and absorption [8]. Recent research progression has led to the re-examination of the key beneficial role of bile acids in health and diseases, including *Clostridium difficile* infection [9], NE [2], campylobacteriosis [10], and longevity [11]. Bile acids are the byproducts of cholesterol metabolism. Bile acids synthesized from cholesterol and conjugated in human hepatocytes are stored in the gallbladder and secreted in the intestine as the main primary bile acids of the conjugated forms of tauro- or glyco-cholic acids (T-CA or G-CA) and chenodeoxycholic acids (T-CDCA or G-CDCA) [12]. Bile acids are then bio-transformed in the gut through four distinct pathways: the deconjugation, dehydroxylation, dehydrogenation, and epimerization of the cholesterol core [13].

The early step of bile acid metabolism is deconjugation, which is mediated by bile salt hydrolase (BSH) from intestinal microbiota [14]. BSH (EC 3.5.1.24) catalyzes the choloylglycine hydrolysis of amide bonds in conjugated bile acids to release amino acid of taurine or glycine [15]. The optimal pH of most BSHs is in an acidic range of 4.5–6.0 [16]. The molecular weights of the BSH are variable, ranging from 28 to 50 kDa [17]. BSHs are mainly present in the bacterial cytoplasm as homotetrameric proteins, with a minority of extracellular and other oligomeric forms [18]. Intracellular BSHs have been investigated in various bacteria, such as *C. perfringens*, *Listeria monocytogenes*, *Lactobacillus*, and *Bifidobacterium* species [19].

Despite most BSHs show a similar overall topology, they have different catalytic efficiencies and substrate specificities. Three-dimensional structures of the BSH enzymes were reported from the five bacteria: *Bifidobacterium longum* [20], *Lactobacillus salivarius* [21], *Enterococcus faecalis* [22], *C. perfringens* [16], and *Bacteroides thetaiotaomicron* VPI-5482 [23]. The highest BSH activity among bacteria isolated from chickens were from *Enterococcus faecium* and *C. perfringens* [24]. The acyltransferase activity of bile salt hydrolase/transferase (BSH/T) in *C. perfringens* rapidly performed an acyl transfer with the available amino acid (all proteinaceous amino acids except proline and aspartate) at an optimal pH of 5.3 [25].

Besides deconjugation, the bile acids are also transformed by dehydroxylation, dehydrogenation, and epimerization using coenzyme A ligase (CAL), hydroxysteroid dehydrogenase (HSDH), dehydratase (DH), oxidoreductase (R), and coenzyme A hydrolase (CAH). *Clostridium scindens* encodes the *bai* operon (CAL, R, DH, HSDH, CAH) for the sequential transformation of CA or CDCA to intermediate bile and secondary bile acid deoxycholic acid (DCA) or lithocholic acid (LCA) [26]. Most intestinal bile acids (~95% in humans) are absorbed and recirculated back to the liver and gallbladder through the portal vein in a process of enterohepatic recirculation. The bile acid pool is relatively constrained, and a dietary supplementation of DCA increases DCA but reduces CDCA levels in chicken ileum [4]. Dietary DCA prevented clinical and subclinical NE and restored the reduced ileal total bile acid level in NE birds [2,4,27]. In a cage experiment, we observed that most ileal DCA existed in a conjugated form of taurodeoxycholic acid (TDCA) [28], although *C. perfringens* expressed BSH.

Based on the abovementioned knowledge, in this study, we aimed to deconjugate TDCA into DCA by expressing secretory BSH to increase the potency of TDCA against *C. perfringens*. We cloned and expressed BSH in *Escherichia coli* and *Bacillus subtilis*. The results showed that deconjugating TDCA by the recombinant BSH increased its efficacy in inhibiting *C. perfringens* growth. The findings could be used for designing new strategies against *C. perfringens*-induced chicken NE and relevant enteritis.

## 2. Materials and Methods

### 2.1. Bacterial Strains, Media, and Growth Condition

*E. coli* DH 5α (Thermo Fisher Scientific at Waltham, MA, USA) was used for plasmid construction, and *E. coli* BL21 (Thermo Fisher Scientific at Waltham, MA, USA) was used for protein overexpression. *E. coli* transformants were selected on Luria Bertani (LB, Genesee Scientific, Morrisville, NC, USA) broth or agar plates supplemented with 100 μg/mL ampicillin (Alfa Aesar, Ward Hill, MA, USA) or 100 μg/mL kanamycin (IBI Scientific, Dubuque, IA, USA), depending on the plasmid resistant gene. *Lactobacillus johnsonii* (ATCC33200, ATCC, Manassas, VA, USA,) and Bifidobacterium longum (ATCC BAA-999, ATCC, Manassas, VA, USA,) were cultured on de Man–Rogosa–Sharpe agar (MRS, BD, Franklin lakes, NJ, USA) and Tryptic Soy agar (*TSA*, Criterion, Santa Maria, CA, USA) plates, respectively.

To visualize the BSH activity, *E. coli*, *B. longum*, and *L. johnsonii* were cultured at 37 °C on TSA *or* MRS (*L. johnsonii* only) plates with 3.7 mM TDCA (Alfa Aesar, Ward Hill, MA, USA) and 2.2 mM calcium chloride (CaCl_2_) (Fisher Scientific, Fair lawn, NJ, USA) for 48 h under anaerobic conditions or 10% CO_2_ air for *L. johnsonii*. Plasmid pET-28a(+) was purchased from Thermo Fisher Scientific at Waltham, MA, USA.

*Bacillus subtilis* strain 168 1A1 and integration plasmid pDR111 were obtained from the *Bacillus* Genetic Stock Center (BGSC) at Ohio State University. *C. perfringens* strain CP1, isolated from our previous chicken studies, was verified positive for various toxins, including cpa, cpe, and netB [29,30]. The *C. perfringens* strains were grown in Brain–Heart Infusion (BHI, Legacy Biologicals, Mt Prospect, IL, USA) or TSA supplemented with sodium thioglycolate (BD, Franklin Lakes, NJ, USA) and sheep blood (Quad Five, Ryegate, MT, USA) with or without antibiotic D-cycloserine (TCI, Tokyo, Japan).

### 2.2. Bile Acid Inhibition of C. perfringens Growth and Hydrogen Sulfide (H_2_S) Production

*C. perfringens* CP1 cultured for 24 h on no-antibiotic TSA plates was inoculated into 1 mL BHI in the presence of TDCA and DCA at 0, 0.2, 0.5, or 1 mM. The bacterium was cultured for 24 h under anaerobic conditions and its growth was measured by colony forming unit (CFU) enumeration using serial dilution and plating on TSA plates with D-cycloserine.

For H_2_S production, filter papers (VWR, West Chester, PA, USA) saturated with lead acetate (Pb(OAc)_2_) (Ward’s Science, Rochester, NY, USA), were dried, autoclaved, and placed on the cap of the tubes. The bacteria were cultured under anaerobic conditions at 37 °C. After 24 h, H_2_S production was visualized and imaged. H_2_S production was confirmed when the paper color was changed from white to brown. A rotten egg stink could be smelled in tubes with brown papers. The darker the color, the more H_2_S production and stink odor, as described in the earlier study [29].

### 2.3. Bacterial RNA Extraction and Gene Expression Using Real-Time PCR

*C. perfringens* CP1 was inoculated in 1 mL of BHI broth in the presence of TDCA or DCA (Alfa Aesar, Ward Hill, MA, USA) at 0 and 0.5 mM and was cultured under anaerobic conditions. After 4 h, the cells were collected, and total RNA was extracted using TRIzol (Thermo Fisher Scientific, St. Louis, MO, USA) as described earlier [4,30]. After cDNA was synthesized using M-MLV (NE Biolab, Ipswich, MA, USA) and a random hexamer (NE Biolab, Ipswich, MA, USA) [31], the virulence gene expression of *asrA1*, *netB*, *colA*, and *virT* was determined using SYBR Green PCR Master Mix (Bio-Rad, Hercules, CA, USA) in a Bio-Rad 384-well real-time PCR (qPCR) system (Bio-Rad, Hercules, CA, USA) as described in the previous paper [28]. The primer sequences of *gyrA*, *asrA1*, and *virT* genes were described previously [28] and here were the primer sequences: *netB*_forward: 5′-ggaaaaatgaaatggcctga-3′; *netB*_reverse: 5′-gcaccagcagtttttccttc-3′; *colA*_forward: 5′-taggaacaaaggcgcaagat-3′; *colA*_reverse: 5′-gaatactgcattccccttgc-3′. The gene expression of the fold-change was calculated using the ΔΔCt method [31] and *gyrA* as an internal control.

### 2.4. Clone and Construct bsh Gene into E. coli Plasmid

The genomic DNA of *B. longum* was extracted using bead beater disruption and phenol/chloroform (VWR, Solon, OH, USA) separation as described previously [10]. The *bsh* gene was amplified using a Bio-Rad T100 PCR thermal cycler (Bio-Rad, Hercules, CA, USA). The following primers appended with BamHI and BmtI restriction sites were used: *Bl-bsh*_forward (from *B. longum*): 5′-cgcggatcctcgggcgacgctgatgag-3′ and *Bl-bsh*_reverse: 5′-ggtgctagcatgtgcactggtgtccgt-3′. The PCR reactions were performed according to the manufacturer’s recommendations. The PCR program consisted of an initial step at 95 °C for 3 min, followed by 35 cycles of 95 °C for 30 s, 60 °C for 60 s, and 68 °C for 30 s, and a final step at 68 °C for 5 min. Subsequently, the plasmid pET-28 and the PCR products were digested with BamHI and BmtI enzymes (all restriction enzymes from NE Biolab, Ipswich, MA, USA) and ligated. The resultant plasmid was named pET-BSH.

### 2.5. Transform Competent E. coli Cells

The transformation process for heat-shock competent *E. coli* DH 5α involved incubating 60 µL cells with 100 ng of pET-BSH for 45 min on ice, followed by a 90-s heat shock at 42 °C. Subsequently, the cells were placed on ice for 2 min, and 1 mL of LB medium was added. The cells were then incubated with shaking at 37 °C for 1 h. The cells were centrifuged, and the pellet was resuspended and plated on LB plates supplemented with 100 µg/mL kanamycin. pET-BSH was isolated using the QIAprep Spin Miniprep Kit (QIAGEN, Hilden, Germany).

For the transformation of electroporation competent *E. coli* BL21, 100 ng of pET-BSH was incubated with 60 µL cells for 10 min, then transferred to an icy cuvette. An electric shock was applied to the cuvette at 2500 volts for 5 milliseconds. Moreover, 1 mL of LB was then added to the cuvette, and the cells were incubated with shaking at 37 °C for 1 h. The cells were centrifuged, and the cell pellet was resuspended and plated on 100 µg/mL kanamycin LB plates.

### 2.6. Overexpress and Purify BSH Protein

The BL21 cells, carrying either pET-BSH or empty pET28, were grown on LB broth until reaching 0.5 optical density at 600 nm (OD_600_). A final concentration of 0.4 mM of isopropyl β-D-1-thiogalactopyranoside (IPTG) (TCI, Tokyo, Japan) was added to the bacterial culture, and then the cells were incubated at 37 °C. After 3 h of incubation, the BL21 cells carrying the overexpressed His-tag BSH protein were collected by centrifugation. The cell pellet was disrupted and the BSH was purified using Ni-NTA agarose of immobilized metal ion affinity chromatography (IMAC) with the HisLink Spin Protein Purification System (Promega in Madison, WI, USA). Briefly, 700 µL of the bacterial culture was transferred to a 1.5 mL tube, and 70 µL of FastBreak Reagent/Dnase was added to lyse the cells. Ni-NTA agarose beads were added to the 1.5 mL tube and incubated with shaking for 30 min. The beads were then transferred to a spin column and washed with binding/washing buffer for three times. The BSH on the beads was eluted in 200 µL elution buffer. The eluted BSH was detected by an anti-His antibody (Genscript, Piscataway, NJ, USA). 

### 2.7. Subclone, Transform, and Express Secretory bsh Gene in B. subtilis

To insert His-tags into shuttle plasmid pDR111, two His-tag oligonucleotides (His _forward: 5′-gatctcagtggtggtggtggtggtg-3′ and His_reverse: 5′-gatccaccaccaccaccaccactga-3′) were synthesized from IDT (Coralville, IA, USA) to destroy the right but keep the left BamHI site. The two oligonucleotides were annealed in a PCR machine programming the initial step at 95 °C for 2 min, followed by 1.5 °C drop/cycle/min for 50 cycles and finally 20 °C. The annealed oligonucleotides were phosphorylated at the 5′ end and ligated with pDR111 digested with BamHI and dephosphorylated at the 5′ end. The resultant plasmid is named pDR111-His. The *bsh* gene from *B. longum* was amplified through PCR using the pET-BSH as a template DNA. The 951 bp *bsh* fragment containing two restriction sites for BamHI and BmtI was digested and ligated into the pDR111-His digested with the two enzymes, resulting in plasmid pDR111-His-BSH. The promoter and signal peptide (SP) regions of the *B. subtilis AmyE* gene were amplified through PCR with primers *AmyE*_forward: 5′-tgcaagcttaacaaaattctccagtcttcacatcg-3′ and *AmyE*_reverse: 5′-atgctagcaattcagcactcgcagcc-3′ using the pDR111 vector as a template DNA. The 228 bp *AmyE* SP fragment containing restriction sites for BamHI and HindIII was digested and ligated with pDR111-His-BSH digested with the two enzymes, resulting in plasmid pDR-BSH. pDR-BSH was then transformed and amplified in *E. coli* DH 5α growing in 100 µg/mL ampicillin LB. pDR-BSH was isolated using the QIAprep Spin Miniprep Kit.

For transforming pDR-BSH into *B. subtilis*, a modified high-osmolarity protocol was used. Briefly, a single colony from *B. subtilis* 168 1A1 was selected and cultured in LB with 0.5 M sorbitol (TCI, Tokyo, Japan). After 24 h, 100 µL *B. subtilis* was plated on a 0.5 M sorbitol LB plate for 6 h. The cells were scrapped from the plates and underwent four washes with ice-cold 0.5 M sorbitol and 0.5 M mannitol (Sigma, St. Louis, MO, USA). After centrifugation and resuspension, 60 µL of the cells was promptly subjected to electroporation transformation with 100 ng pDR-BSH. The transformed cells were grown in 0.5 M sorbitol and 0.38 M mannitol LB broth at 37 °C for 30 min. After centrifugation and resuspension, the pellet was plated on 100 µg/mL spectinomycin LB plates, resulting in *B. subtilis* with pDR-BSH (*B-sub*-BSH).

### 2.8. Evaluate the Expressed BSH with Dot-Blot, Coomassie Blue Staining, and Western Blotting

The BSH protein concentrations from BL21 or B. subtilis were measured by Bio-Rad assay as described before [30]. For the Dot-Blot assay, 2 µL sample (2 μg/µL) was dropped onto a nitrocellulose membrane (GE HealthCare, Chicago, IL, USA). After overnight drying in room temperature, the blot was blocked in 5% bovine serum albumin (*BSA*, Sigma, St. Louis, MO, USA) of 0.05% Tween 20 (J.T.Baker, Phillipsburg, NJ, USA) TBST buffer. The membrane was incubated with 1:1000 dilution of His-tag antibodies in blocking solution overnight at 4 °C. After washing, the membrane was incubated with a 1:2000 dilution of HRP-conjugated donkey anti-rabbit secondary antibody (GE HealthCare, Chicago, IL, USA) in blocking solution for 30 min at room temp. Proteins were detected by enhanced chemiluminescence (ECL) reagents (Revvity Health Sciences, San Francisco, CA, USA). The results were imaged using the Odyssey Fc Imaging System (LI-COR Biosciences, Lincoln, NE, USA).

The expressed proteins were also analyzed by SDS–polyacrylamide gel electrophoresis (SDS-PAGE), followed by Coomassie blue staining. Briefly, after electrophoresis and washing three times, the gel was transferred to a gel box, submerged in Coomassie blue (Thermo Fisher Scientific, St. Louis, MO, USA), and stained for 2 h with shaking. After washing, the gel was de-stained for 1 h or until a clearer gel background was attained. For the Western blot, 20 μg/lane of protein samples were separated on a 10% SDS-PAGE gel, transferred onto a nitrocellulose membrane, and blocked in 5% BSA TBST buffer. The membrane was incubated with 1:1000 dilution of His-tag antibodies in blocking buffer overnight at 4 °C. After washing, the membrane was incubated with 1:2000 diluted secondary antibody and images were taken. The density of Western Blot bands and Dot-Blot as evaluated using ImageJ and presented as relative quantification (version-1.54i, NIH, Bethesda, MD, USA).

### 2.9. C. perfringens Growth Inhibition Assay with B-sub-BSH

To evaluate the efficacy of the secretory BSH, *C. perfringens inhibition assay was conducted* with TDCA deconjugation by *B-sub-*BSH. Briefly, *B-sub-*BSH was pre-cultured with 0, 0.5 and 1 mM TDCA in BHI broth for 24 h under anaerobic conditions. After the *B-sub-*BSH pre-culture media were autoclaved, *C. perfringens* was inoculated and incubated at 37 °C under anaerobic conditions. After 24 h, *C. perfringens* growth was enumerated by serial dilution and plating on the selective TSA plates.

### 2.10. Statistical Analysis

One-way ANOVA was applied to compare means among multiple groups of treatments. Multiple comparisons of Fisher’s LSD test using Prism 7.0 software (GraphPad Software, Franklin Street, FL, USA) were then conducted. Experiments were considered statistically significant if *p*-values were <0.05.

## 3. Results

### 3.1. Conjugated TDCA Was Less Potent against C. perfringens Virulence Compared to DCA

**Figure 1:**
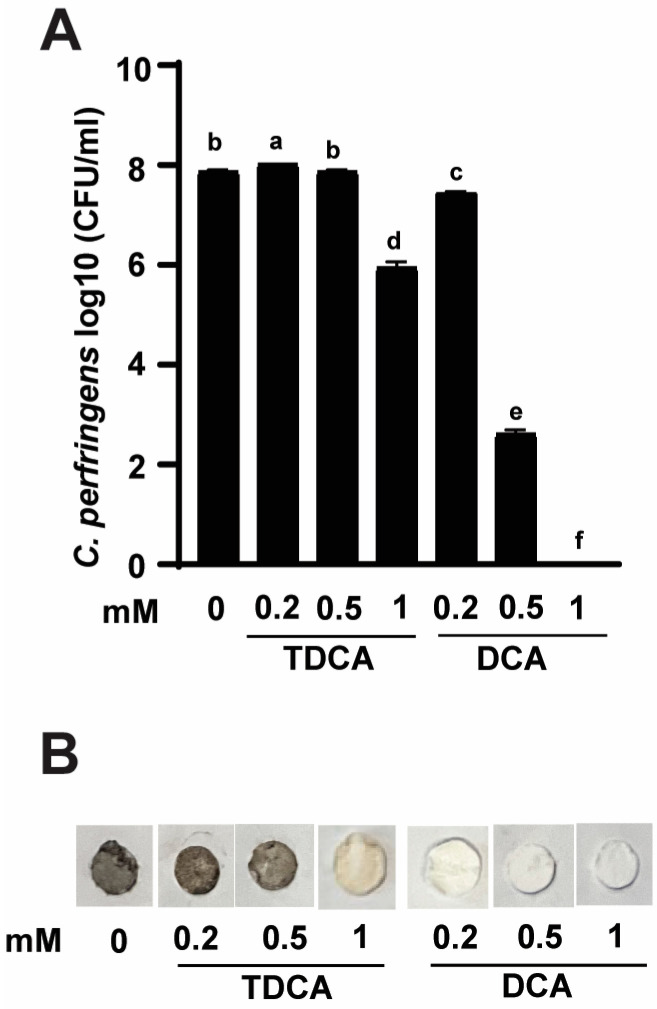
Conjugated TDCA was less potent against *C. perfringens* virulence compared to conjugated DCA. *C. perfringens* was cultured with various concentrations of TDCA and DCA for 24 h under anaerobic conditions. (**A**) *C. perfringens* enumeration by serial dilution and plating. (**B**) Detection of H_2_S production via lead acetate discs. The group with 0 mM bile acids served as the control. Results were representative of 3 independent experiments. All graphs show mean + SEM. Different letters (a–f) indicate *p* < 0.05.

To determine if deconjugated DCA was more effective against *C. perfringens* virulence than the conjugated TDCA form, *C. perfringens* was grown in varying concentrations of TDCA or DCA. After 24 h, *C. perfringens* growth reached 7.8 log10 CFU/mL in the control group with 0 mM bile acids. TDCA at 1 mM significantly reduced *C. perfringens* growth by 1.8 log10 CFU/mL compared to the control, possibly because the pathogen expressed BSH and could deconjugate TDCA into DCA and inhibit its own growth. Notably, 0.2, 0.5, and 1 mM DCA significantly reduced *C. perfringens* growth by 0.4, 5.2, and 7.8 log10 CFU/mL, respectively, compared to the control (Figure 1A), suggesting that TDCA is less potent than DCA against *C. perfringens* growth.

DCA reduced rotten-egg smell gas in NE birds and in in vitro experiments [29]. Because TDCA was less inhibitory to *C. perfringens* growth compared to DCA, we hypothesized that TDCA might be less effective to reduce *C. perfringens* virulence, such as the production of the foul-smell H_2_S. We then conducted an H_2_S detection assay using paper discs saturated with lead acetate. Consistently, *C. perfringens* culture characterized by rotten-egg smell and changed lead acetate disc paper into dark brown color in the control group of 0 mM bile acids (Figure 1B). Notably, consistent with the difference in *C. perfringens* growth inhibition, TDCA was less effective in reducing the foul smell and the dark brown discs compared to DCA.

### 3.2. TDCA Was Less Potent against C. perfringens Virulence Gene Expression Compared to DCA

**Figure 2:**
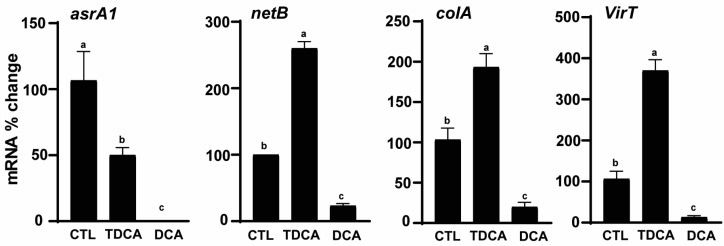
TDCA was less potent against *C. perfringens* virulence gene expression compared to DCA. *C. perfringens* CP1 was cultured with control BHI (CTL), TDCA, and DCA at 0.5 mM for 4 h. After RNA extraction and cDNA reverse transcription, the accumulation of virulence genes was quantified by real-time PCR (qPCR). Results were representative of three independent experiments. All graphs show mean + SEM. Different letters (a–c) indicate *p* < 0.05.

The differential reduction in *C. perfringens* growth and H_2_S production between DCA and TDCA could be mediated at a transcriptional or translational level. Because of lacking antibodies against most *C. perfringens* proteins, it was more feasible to measure its transcription activities. Consistent with the lead disc assay, the expression of H_2_S producing gene *asrA1* was reduced by 53 or 100% by 0.5 mM TDCA or DCA, respectively, compared to the control BHI (CTL) (Figure 2). Notably, NetB toxin gene *netB* was reduced by 77% with 0.5 mM DCA compared to CTL, while it was increased in the presence of 0.5 mM of TDCA by 160%. Moreover, the kappa toxin gene *colA* accumulation was reduced by 0.5 mM DCA at 81% compared to CTL, while 0.5 mM TDCA increased the gene by 87%. The regulatory RNA gene *virT* was reduced by 88% with DCA compared to CTL, while TDCA increased the gene by 247%. These results suggest that TDCA was less effective than DCA in reducing H_2_S production and other virulence activities at the transcriptional level.

### 3.3. Clone and Overexpress of BSH in E. coli Strains DH5α and BL21

**Figure 3:**
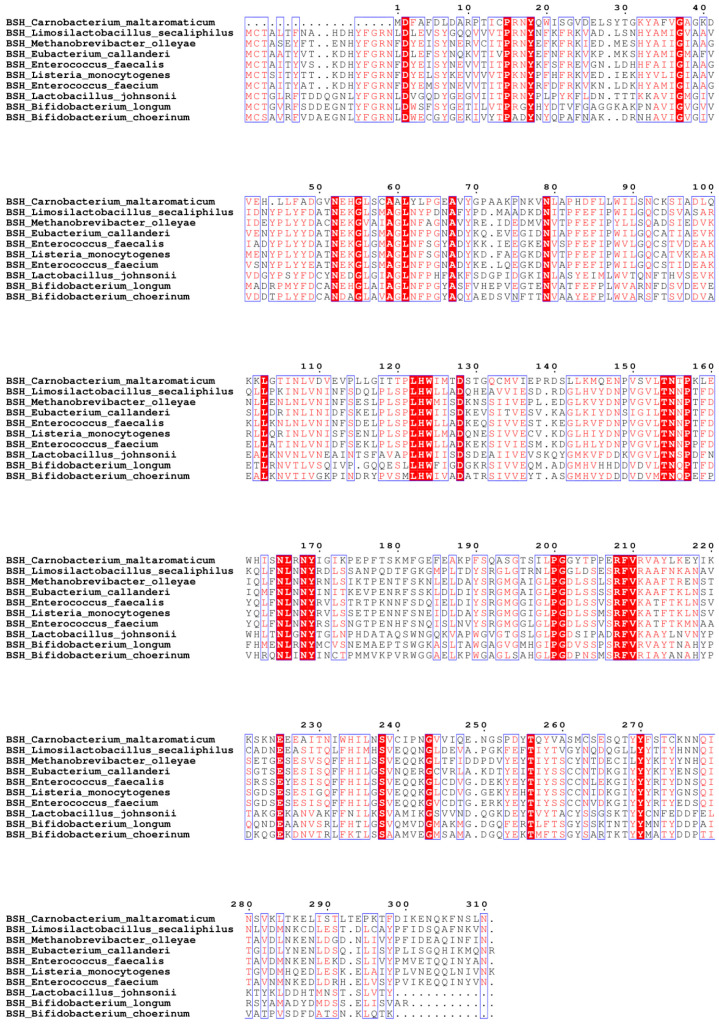
The *bsh* gene alignment. It presents a multiple sequence alignment of amino acid sequences from bile salt hydrolase (BSH) in ten different bacterial species. Identical amino acids are highlighted with a red background, similar amino acids in a group are shown in red letters, and similar amino acids across groups are shown in blue frame.

**Figure 4:**
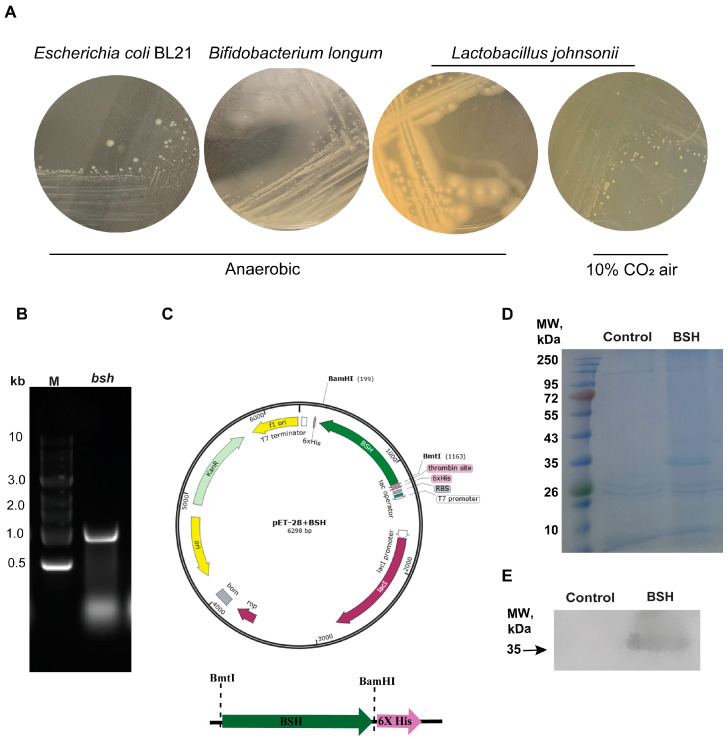
Clone and overexpress BSH in *E. coli* BL21. (**A**) *E. coli*, *B. longum*, and *L. johnsonii* were cultured with 3.7 mM TDCA and 2.2 mM CaCl_2_ for 48 h under anaerobic conditions or 10% CO_2_ air for *L. johnsonii*. (**B**) Agarose gel electrophoresis image showing PCR product of *bsh* gene from *B. longum*. (**C**) The plasmid map for the constructed pET-BSH is depicted. (**D**) Coomassie blue staining for SDS-PAGE gel of transformed *E. coli* BL21 lysate. (**E**) Western blot analysis of purified BSH from *E. coli* BL21 using anti-His-tag antibody.

Because conjugated bile acids, such as TCA or TDCA, are deconjugated by microbial enzyme BSH [32], we then conducted molecular experiments of cloning and expressing the *bsh* gene for producing recombinant BSH. We aligned ten bacterial *bsh* genes (Figure 3). Among them, *L. johnsonii* and *B. longum* shared more sequences compared to other bacteria. Based on the sequence alignment and literature review, we used *L. johnsonii* and *B. longum* to amplify *bsh* genes. To visualize BSH activity, we used plates supplemented with TDCA and CaCl_2_. The plates images are shown in Figure 4A, with a DCA precipitation halo around the BSH-positive colonies of anaerobic *L. johnsonii* and *B. longum*, while there was no halo in anaerobic *E. coli* and *L. johnsonii* colonies under 10% CO_2_ air. The results suggest that BSH is susceptible to oxygen inactivation. In this manuscript, we only show *bsh* cloning work using *B. longum*.

The *bsh* gene from *B. longum* genomic DNA was PCR-amplified using primers appended with BamHI and BmtI restriction sites. The PCR product was loaded and separated into 1% agar gel and then imaged (Figure 4B). The *bsh* PCR gel band around 1 kb was excised and purified using the Qiagen Gel Purification Kit. After BamHI and BmtI digestion and ligation, *bsh* was inserted into pET-28a and was chemically transformed into *E. coli* DH5α cells. The resultant plasmid was named pET-BSH, and an overview map of the plasmid is shown in Figure 4C.

After amplification in DH5α and purification by the Qiagen Mini Plasmid Kit, pET-BSH was electroporation-transformed into *E. coli* BL21 cells for protein overexpression. The crude protein was subjected to SDS PAGE gel and Coomassie blue staining. As shown in Figure 4D, a clear band at around 35 kDa was observed in the crude lysate of BL21 transformed with pET-BSH, and the size was in accordance with BSH from *B. longum*. BSH was further purified with Ni-NTA agarose of IMAC and was evaluated using Western blot analysis and anti-His antibodies. As shown in Figure 4E, a significantly clear band at around 35 kDa was observed in the BSH blot compared to the control (134.3 ± 0.48 vs. 1.0 ± 0.48).

### 3.4. Clone and Express Secretory BSH in B. subtilis Strain 168

**Figure 5:**
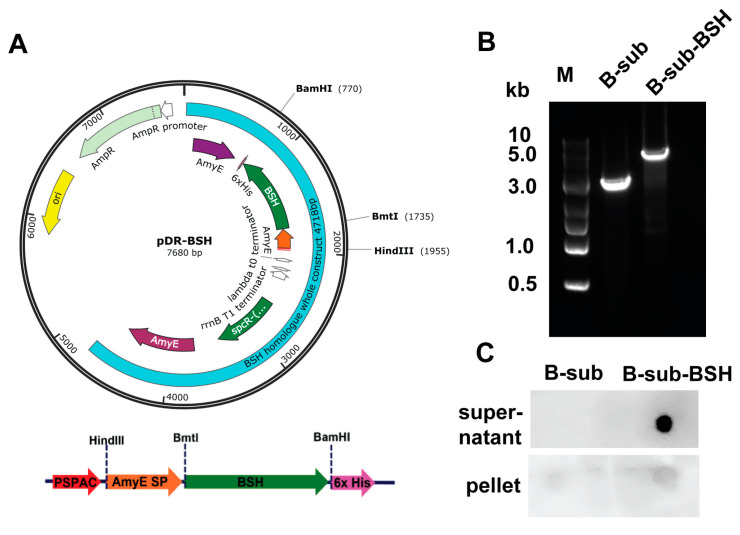
Clone and express secretory BSH in *B. subtilis*. (**A**) Plasmid map for constructed pDR-BSH that contains *spcR*, promoter, secretory signal peptide (SP) regions of *AmyE* gene, *bsh* gene from *B. longum*, and His-tag. (**B**) Agarose gel electrophoresis image showing PCR product of inserted combined *bsh* construct inside genomic *B. subtilis*. (**C**) Dot-Blot analysis of purified protein from *B-sub* and *B-sub*-BSH for both supernatants and pellets.

BSH is very sensitive to oxygen [32] and is an intracellular protein enzyme present in most bacteria, including *Bifidobacterium* species [19]. We also showed the inactivation of BSH in *L. johnsonii* under 10% CO_2_ air in Figure 4A. Hence, it would be impractical to directly use the BSH protein in feed due to the inactivation of BSH in air. To address these issues, we then conducted a subcloning of the *bsh* gene to *B. subtilis*, a facultative bacterium [33]. We acquired *B. subtilis* strain 168 1A1 and integration shuttle plasmid pDR111 from *Bacillus*. To make secretory BSH, we cloned the sequence of the promoter and SP regions of the *AmyE* gene and named it *AmyE-SP*. *bsh* and *AmyE-SP* were assembled using overlapping PCR with HindIII and BamHI restriction sites (Figure 5A). The resultant *AmyE-SP-bsh* and pDR111 were digested with HindIII and BamHI, ligated, and transformed into *E. coli* DH5α cells for amplification. The resultant plasmid was named pDR-BSH, and an overview map of the plasmid is shown in Figure 5A. pDR-BSH was then electroporation-transformed into *B. subtilis* 168 1A1. The resultant bacterium was named *B. subtilis*-BSH (*B-sub*-BSH). To confirm the integration of the *AmyE-bsh-SP-AmyE-spcR-AmyE* sequence (light-blue region on the right, ~4.7 kb) into the genome, PCR was performed, and the product was run on gel and imaged. As shown in Figure 5B, the PCR product of wild-type *B-sub* was around 3 kb and that in the transformed *B-sub*-BSH was around 5 kb.

To confirm that BSH was secreted, supernatants and cell pellets of *B-sub* and *B-sub*-BSH were collected. The proteins from the supernatants and pellets were evaluated using Dot-Blot analysis using anti-His antibodies. As shown in Figure 5C, a significantly strong dot was observed in the *B-sub*-BSH supernatant compared to B-sub (507.8 ± 0.60 vs. 1.0 ± 0.60), while the B-sub-BSH pellet also showed a significant dark dot compared to the B-sub pellet (507.8 ±0.60 vs. 1.0 ± 0.60). From these results, we concluded that we cloned secretory BSH into *B-sub*-BSH.

### 3.5. B. subtilis-BSH Deconjugated TDCA against C. perfringens Growth

**Figure 6:**
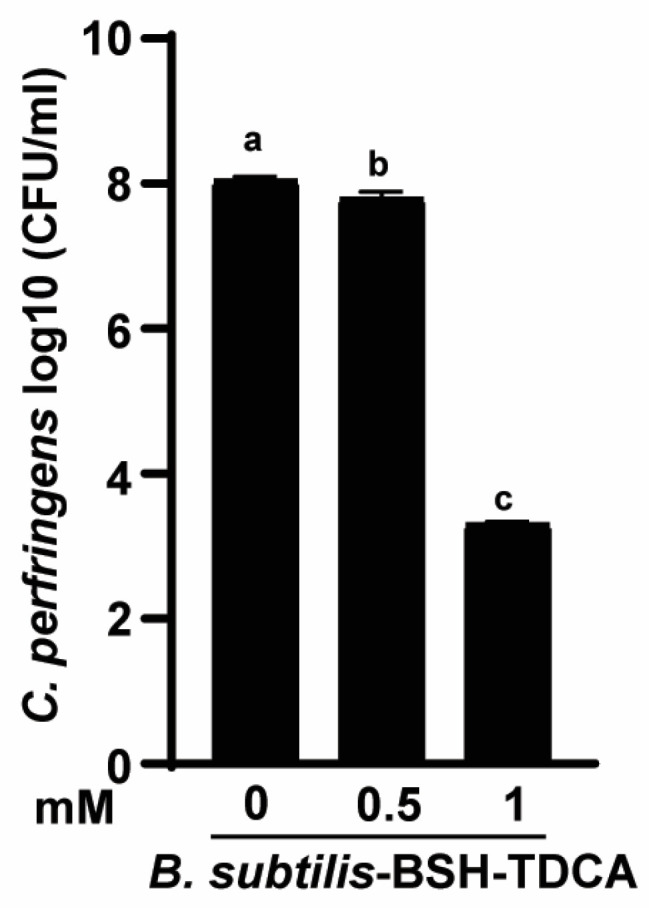
*B. subtilis*-BSH deconjugated TDCA against *C. perfringens* growth. *C. perfringens* was pre-cultured with 0, 0.5, and 1 mM TDCA and *B-sub*-BSH for 24 h under anaerobic conditions. After autoclave, *C. perfringens* CP1 was inoculated and incubated for 24 h and its growth was enumerated by plating. Results were representative of three independent experiments. All graphs show mean + SEM. Different letters of a–c mean *p* < 0.05.

Despite the Dot-Blot assay of the secretion of BSH by *B-sub*-BSH, it was imperative to evaluate the enzyme activity of the secretory BSH. To address this concern, we performed a *C. perfringens* growth inhibition assay using pre-cultured *B-sub*-BSH and TDCA. As shown in Figure 6, *C. perfringens* in the control group of *B-sub*-BSH and 0 mM TDCA grew to 8.1 log10 CFU/mL. Interestingly, the group of *B-sub*-BSH and 0.5 mM TDCA only reduced *C. perfringens* growth by 0.5 log10 CFU/mL compared to the control. Importantly, *B-sub-*BSH and 1 mM TDCA reduced *C. perfringens* growth by 4.76 log10 CFU/mL compared to the control, representing a greater than 59% log10 CFU/mL reduction.

## 4. Discussion

Although dietary DCA effectively reduced severe clinical [2,27] and subclinical NE [4] in previous studies, we found that most DCA in the ileum of birds raised in a cage pen study existed in a conjugated form of TDCA [23]. Consistent with the notion of less efficacy of conjugated vs. deconjugated bile acids against bacterial pathogens, in this study, we found that TDCA was less potent against *C. perfringens* growth and virulence compared to DCA. Similarly, TCA was less efficient than CA in inhibiting *C. perfringens* growth [2]. Germ-free or antibiotics-treated mice carrying mostly conjugated bile acids are susceptible to *Clostridium difficile* infection, and bio-transforming secondary bile acids (DCA and LCA) are some of the key protective functions of the microbiota [9]. DCA reduces *C. difficile* vegetative growth in a dose-depended manner and represses the main *C. difficile* toxin *tcdB* production [34]. DCA also reduces *C. difficile* sporulation efficiency through downregulating *spo0A* expression. We found that DCA effectively reduced *C. perfringens* toxin gene expressions of *asrA1*, *netB*, *colA*, and *virT*. Interestingly, both conjugated and deconjugated bile acids directly bind to and inhibit TcdB activity and protect human lung fibroblast IMR-90 cells against the TcdB-induced cell toxicity of roundness dose-dependently [35], suggesting that the level of total bile acids pools is also important in *C. difficile* infection. Because of the closeness between *C. difficile* and *C. perfringens*, it is important to elucidate whether and how bile acids directly bind and inhibit *C. perfringens* toxins and their induction of chicken NE.

Conjugated bile acids increase *C. perfringens* virulence. *C. perfringens* spores are germinated by amino acids and conjugated bile acids such as TCA, TCDCA, and TDC [36]. One of the early steps of bile acid metabolism by intestinal microbiota is BSH (EC 3.5.1.24)-mediated deconjugation [14]. BSH (EC 3.5.1.24) catalyzes the choloylglycine hydrolysis of amide bonds in conjugated bile acids to release an amino acid of taurine or glycine [15]. BSHs belong to the N-terminal nucleophilic (Ntn) hydrolase superfamily and share a core αββα structure with an N-terminal catalytic cysteine residue, and additional conserved active sites are Arg18, Asp21, Asn82, Asn175, and Arg228 [16]. A total number of 591 BSHs identified from the Human Microbiome Project (HMP) database are assigned to 117 genera from 12 phyla, such as *Actinobacteria*, *Bacteroidetes*, *Euryarchaeota*, *Firmicutes*, and *Proteobacteria* [37]. The molecular weights of the BSH are variable ranging from 28 to 50 kDa [17]. In this study, we found that the *bsh* gene and protein from *B. longum* were ~35 kDa. The findings are consistent with a previous report that shows that the number of amino acids in BSHs from genera *Clostridium*, *Bifidobacterium*, *Lactobacillus*, and *Enterococcus* are 329, 316, 325, and 326, respectively [37].

BSHs are mainly present in the bacterial cytoplasm as homotetrameric proteins with a minority of extracellular and other oligomeric forms [18]. Intracellular BSHs are studied in *Bacteroides fragilis*, *Bacteroides vulgatus*, *Clostridium perfringens*, *Listeria monocytogenes*, *Lactobacillus*, and *Bifidobacterium* species [19]. Although BSH could be overexpressed to produce a large quantity in *E. coli* BL21, BSH susceptibility to oxidative inactivation [38] prevented this method from practical usefulness. Indeed, we found that *L. johnsonii* did not show any halo in the TDCA plate under 10% CO_2_ air, while a visible halo was present under anaerobic conditions. The finding is consistent with the observation of BSH-positive *L. johnsonii* producing a halo in a TDCA plate under anaerobe [39]. To overcome this issue, we used the *B. subtilis* secretory expression system. *B. subtilis* is a facultative bacterium [33] and should grow well in ileum, where abundant conjugated bile acids are present. We then subcloned the *B. longum bsh* gene into *B. subtilis* integration plasmid pDR111. According to the literature, *Bifidobacterium* BSH is an intracellular protein [19]. We also cloned and inserted the promoter and secretory signal peptide (SP) region from *B. subtilis* amylase into the 5′ end of *bsh*. The secreted BSH protein was validated from the positive results in the supernatant of *B-sub*-BSH in the Dot-Blot assay. Importantly, the functional assay showed that TDCA pre-cultured with *B-sub*-BSH significantly reduced *C. perfringens* growth compared to *B-sub*-BSH only. To apply the findings in the poultry industry, we are planning to feed chickens with *B-sub*-BSH to prevent chicken NE.

An interesting finding in this study is that TDCA itself inhibits *C. perfringens* growth. *C. perfringens* express BSH with activity toward glycine and taurine conjugates of CA [40]. Although the BSH substrate specificity is broad, the K_M_ values for GCDCA and TCA are in the range of 10^−2^ M, whereas those for deoxy-conjugates is generally in the 10^−3^ M range [41]. In a crystal structure study with TDCA, it is showed that *C. perfringens* BSH is a tetrameric N-terminal thiol hydrolase with specific recognition of its cholyl but not of its tauryl product [16]. Interestingly, in our chicken NE experiment, *C. perfringens* did not hydrolyze much TDCA in birds fed dietary DCA [28]. This could have resulted from the self-protection of *C. perfringens* by less hydrolyzation of TDCA to DCA. Similarly, in co-culture, TDCA only partially reduced *C. perfringens* growth compared to DCA. The results suggest the potential to supplement *B-sub*-BSH when fed DCA in feed to prevent chicken NE.

## 5. Conclusions

Our findings are consistent with the notion that conjugated bile acids (e.g., TDCA) were less potent than deconjugated bile acids (e.g., DCA) against bacterial pathogen (e.g., *C. perfringens*) growth and virulence. It could improve the gut health of young animals (e.g., broiler chickens 0 to 60 days of age) through modulating their immature microbiota and bile acid metabolism (e.g., conjugated bile acids). In this study, we developed practically feasible methods to increase the potency of conjugated bile acids using secretory recombinant BSH in *B. subtilis*. Furthermore, the *B. subtilis* secretory expression system could be used to orally deliver other important bioactive proteins such as vaccines and other bile acid enzymes. These findings could be applied to the poultry industry to control chicken NE and other intestinal diseases.

## Data Availability

The data are presented in this paper.
